# India plate angular velocity and contemporary deformation rates from continuous GPS measurements from 1996 to 2015

**DOI:** 10.1038/s41598-017-11697-w

**Published:** 2017-09-12

**Authors:** Sridevi Jade, T. S. Shrungeshwara, Kireet Kumar, Pallabee Choudhury, Rakesh K. Dumka, Harsh Bhu

**Affiliations:** 1 CSIR-4PI, CSIR Fourth Paradigm Institute (Formerly CSIR-CMMACS), Wind Tunnel Road, Bangalore, 560 037 India; 2 GBPNIHESD, GB Pant National Institute of Himalayan Environment and Sustainable Development (Formerly GBPIHED), Almora, India; 3 Institute of Seismological Research, Gandhinagar, India; 4 ML Sukhadia University, Udaipur, India

## Abstract

We estimate a new angular velocity for the India plate and contemporary deformation rates in the plate interior and along its seismically active margins from Global Positioning System (GPS) measurements from 1996 to 2015 at 70 continuous and 3 episodic stations. A new India-ITRF2008 angular velocity is estimated from 30 GPS sites, which include stations from western and eastern regions of the plate interior that were unrepresented or only sparsely sampled in previous studies. Our newly estimated India-ITRF2008 Euler pole is located significantly closer to the plate with ~3% higher angular velocity than all previous estimates and thus predicts more rapid variations in rates and directions along the plate boundaries. The 30 India plate GPS site velocities are well fit by the new angular velocity, with north and east RMS misfits of only 0.8 and 0.9 mm/yr, respectively. India fixed velocities suggest an approximate of 1–2 mm/yr intra-plate deformation that might be concentrated along regional dislocations, faults in Peninsular India, Kachchh and Indo-Gangetic plain. Relative to our newly-defined India plate frame of reference, the newly estimated velocities for 43 other GPS sites along the plate margins give insights into active deformation along India’s seismically active northern and eastern boundaries.

## Introduction

Global Positioning System (GPS) measurements at sites located in the Indian plate and along its seismically active and tectonically complex boundaries provide critical, present-day kinematic boundary conditions for a wide variety of geo-scientific studies, including studies of the tectonics and earthquake cycles of the numerous faults within and along the boundaries of the India plate. Plate tectonic estimates of India plate motion, which are based on reconstructions of the Carlsberg Ridge, seafloor spreading center magnetic anomaly sequences, strikes of transform faults and earthquake slip vectors^1–6^ span millions of years and are thus more useful for studying the long-term evolution of Indian plate tectonics than its present-day deformation.

Previous estimates of India plate motion and deformation internal to the plate with space geodetic observations include^7^ inversion of the velocities of 12 episodic GPS (eGPS) sites located south of 14°N (South India) and Delhi to estimate India plate angular velocities relative to the geodetic reference frame ITRF96 and the Eurasia plate. Their India-Eurasia angular velocity predicted convergence rates along the Himalayan frontal thrust are ~14% slower when compared to the rates predicted by the NUVEL-1A 3-Myr-average India-Eurasia angular velocity. This is consistent with a previous report^8^ of a significant discrepancy between geodetic and geologic estimates of India plate motion. Subsequent estimates of India plate motion have relied increasingly on velocities from continuous GPS sites (cGPS) and eGPS which include: 3 cGPS,ITRF97^9^; 2 cGPS + 4 eGPS located in South Nepal, ITRF2000^10^; 3 cGPS + 2 eGPS located in South Nepal, ITRF2000^11^; 7 cGPS, ITRF2000^12^; 10 cGPS + 2 eGPS, ITRF2000^13^; 4 cGPS, ITRF2005^14^; and 13 cGPS, ITRF2008^15^. All of the above studies are affected to varying degrees by the sparse distribution of GPS sites in the plate interior and often-short observation time spans at many of their GPS sites. In particular, previous authors use few or no GPS sites from western and eastern region, thereby limiting the accuracy with which the India plate rotation pole could be determined.

In this study, we use data from 27 cGPS and 3 eGPS sites from stable regions of the India plate (more than any previous study), including for the first time cGPS sites from western and eastern regions of continental plate interior, to estimate the India plate angular velocity in ITRF2008. The data samples more of the plate interior sites and span more time than any previous study and thus offer the best opportunity to date to establish an upper limit on any deformation internal to the plate. For the first time velocities from seven sites in western India are used for estimation of angular velocity. cGPS observations from another 43 sites located in the Indian subcontinent are used to quantify deformation along the seismically active northern and eastern boundaries of Indian tectonic plate.

## India plate tectonic setting

**Figure 1:**
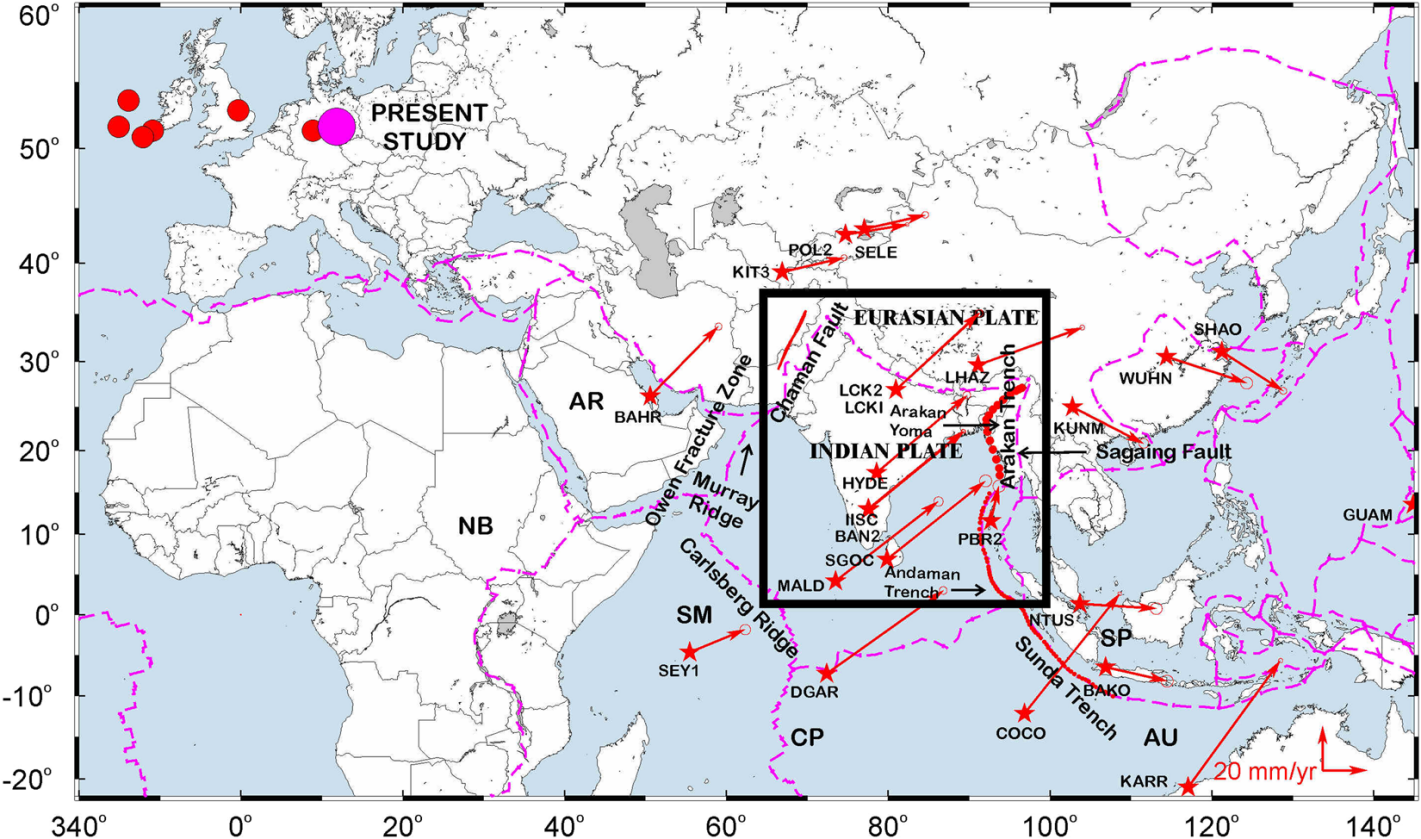
Indian tectonic plate with plate boundaries with IGS sites(stars) and their ITRF2008 velocities tipped with 95% confidence error ellipse. The rectangle shows the region depicted in Fig. 2. India/ITRF pole of rotation of Indian tectonic plate estimated in the present study is plotted along with earlier poles of rotation. AR-Arabia Plate, NB-Nubia Plate, SM-Somalia Plate, CP-Capricorn Plate, AU-Australia plate, SP-Sunda plate. Figure was created using GMT (generic mapping tool) software^72^.

**Figure 2:**
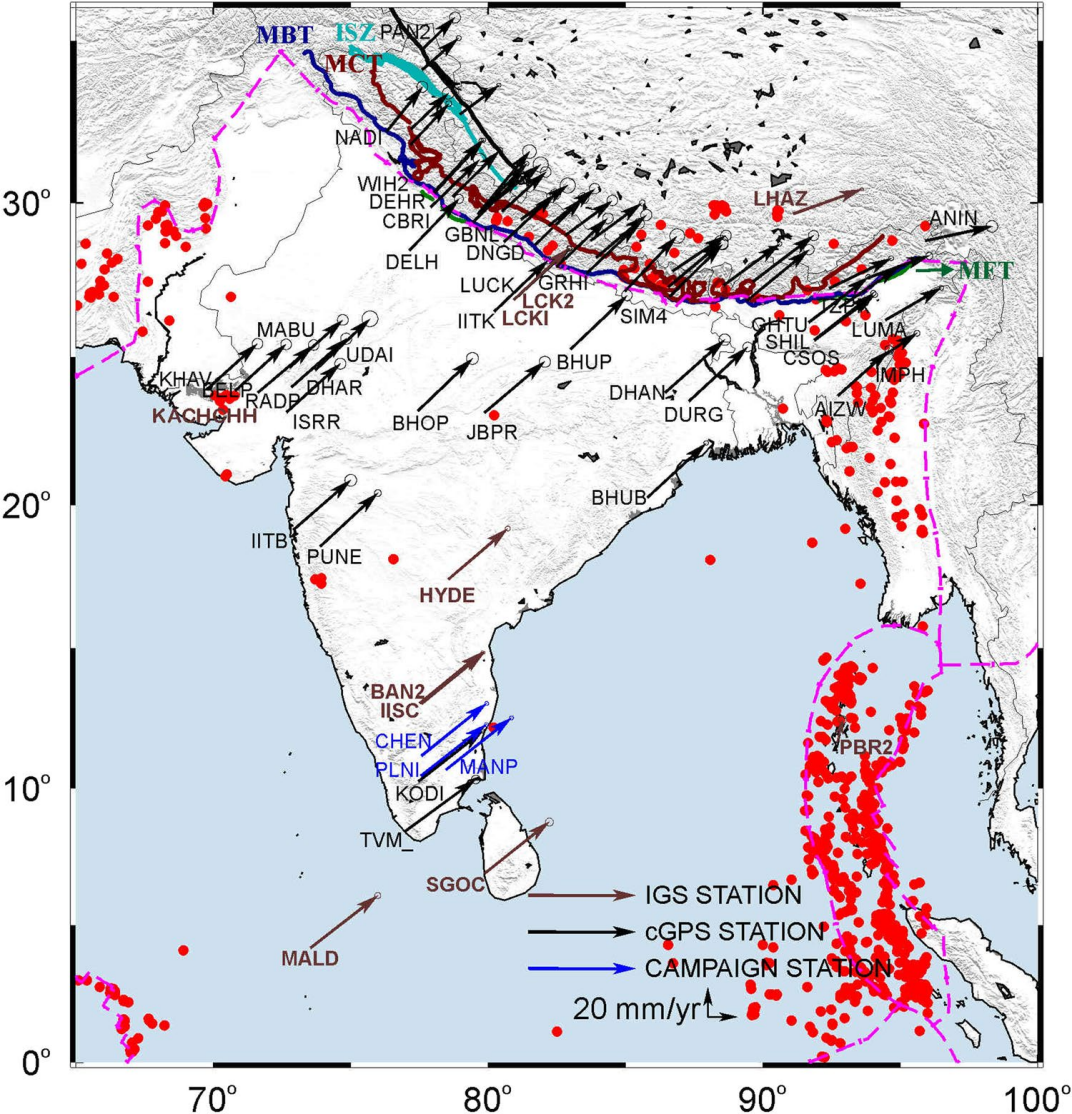
ITRF08 velocities tipped with 95% confidence error ellipse of GPS sites used in this study (Table 1). Earthquakes with magnitude ≥5 from 1970 to 2016 are plotted as solid Red circles (source: http://ds.iris.edu/). Figure was created using GMT (generic mapping tool) software^72^. MFT: Main Frontal Thrust, MBT: Main Boundary Thrust, MCT: Main Central Thrust, ISZ: Indus Suture Zone.

**Figure 3:**
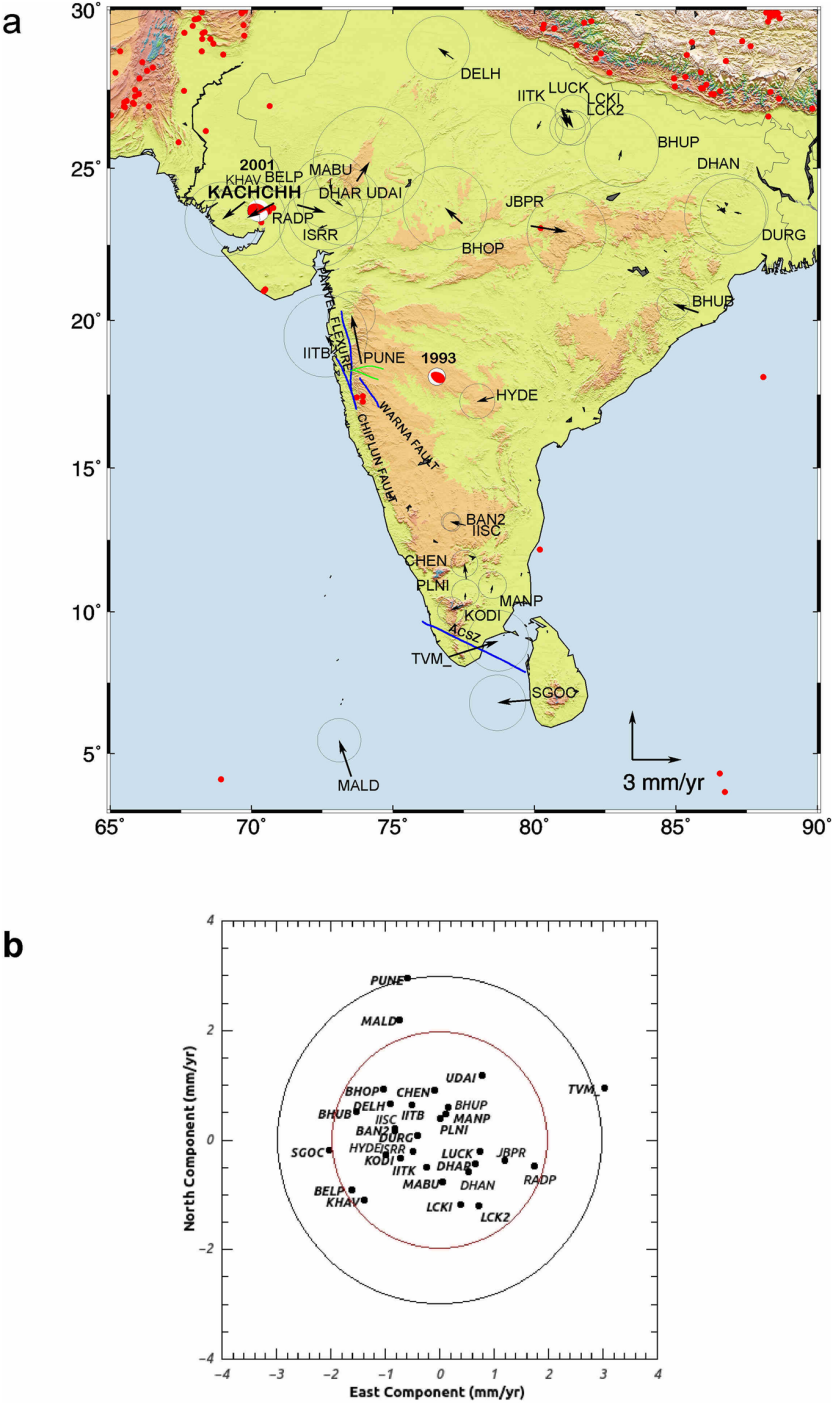
(**a**) Residual velocities tipped with 70% confidence error ellipse of the 30 GPS sites whose velocities were used to estimate the best-fitting India-ITRF2008 angular velocity in Table 2. ACSZ: Achankovil Shear zone, Blue lines are minor lineaments near PUNE GPS site. Focal mechanisms **(**Source: http://www.globalcmt.org/CMT **)** are given for two major earthquakes mentioned in the text, including the 1993 Latur earthquake (Mw 6.3) and the 2001 Bhuj earthquake (Mw 7.6). Earthquakes with magnitude ≥5 from 1970 to 2016 are plotted as solid red circles (source: http://ds.iris.edu/). Figure was created using GMT (generic mapping tool) software^72^. (**b**) North and east residual site velocity components with respect to velocities estimated with the India-ITRF2008 angular velocity of present study given in Table 2. Red and Black circle represent the velocity of ±2 and ±3 mm/yr respectively. Figure was created using qtiplot^73^.

The India plate borders the Eurasia plate on its northern and eastern boundary; Arabian plate on its, western boundary; Somalia, Capricorn, and Australia plates to the south (Fig. 1). Relevant to this study, the ~2500-km-long Himalayan Arc at the northern limit of the India plate accommodates NNE-directed convergence between India and Eurasia, increasing from ~35 mm/yr at the arc’s western end in Kashmir (~75°E) to ~50 mm/yr at its eastern end near the Eastern Himalayan Syntaxis^4^. The western, sinistral shear-dominated plate boundary of Indian tectonic plate consists of the Owen fracture zone and Murray Ridge between India and Arabia^16, 17^ and the Chaman fault zone between the Makran subduction zone and Himalayan Arc^18, 19^. Motion between India and the Sunda Block^20^ (which rotates clockwise with respect to Eurasia) along their dextral, shear-dominated boundary is variously partitioned between convergence along the Andaman and Arakan trenches and structures farther inboard, including the Andaman back-arc spreading center and the Sagaing fault of Myanmar^10^. The 2500 km Himalayan Arc is characterized by several thrust faults that sole into the basal detachment of the Himalayan wedge or the Main Himalayan Thrust which marks the upper boundary of under-thrusting Indian plate^21–24^. Main Frontal Thrust (MFT) marks the southern boundary of the Himalayas and the surface trace of MHT coincides with MFT. Further north (Figs 2 and 5) of it is Main Boundary Thrust (MBT) which marks the southern limit of Lesser Himalaya, Main Central Thrust (MCT) transition between the Lesser and Higher Himalaya, South Tibet detachment (STD) is the transition between Higher and Tethyan Himalaya and Indus Suture Zone (ISZ) is the northern boundary^25, 26^. Major seismic activity (Fig. 2) in the Indian tectonic plate is mostly confined to northern and eastern convergence zones^27, 28^. Seismic activity in peninsular India, except Kachchh region (Figs 2 and 3a), is low with very rare occurrence of earthquakes of magnitude 5.0^29^. Kachchh region recorded several felt earthquakes^30^ throughout 19^th^ century and the notable earthquakes in this region are 1819 Allah bund earthquake^31^, 1956 Anjar^32^ and 2001 Bhuj earthquake^33, 34^. The seismic events in peninsular India are isolated from each other and are mainly due to the movement along the regional dislocations and faults^35–37^.

## Results and Discussions

### India-ITRF2008 angular velocity

Twenty-five continuous and three campaign sites included in our analysis are located on the continental peninsular Indian shield and Indo-gangetic plain and two IGS stations SGOC in Sri Lanka and MALD in the Maldives both located on islands in Indian Ocean (Figs 1, 2 and 3 and Table 1), where previous studies demonstrated that little or no intraplate deformation occurs^7, 12, 13, 15^. We thus estimated the India plate angular velocity in ITRF2008 by minimizing the ITRF2008 GPS velocities of these 30 GPS sites using GLORG. Table 2 gives the pole, angular rotation rate, and covariances for our newly estimated angular velocity, as well as angular velocities estimated by previous authors.

**Table 1:** ITRF and India Fixed rates of GPS sites used in the analysis with the location description and the data span.

Site Code	Lat(^o^N)	Lon(^o^E)	Mode epoch	ITRF08 Velocities				India fixed velocities				Description
				N	σN	E	σE	N	σN	E	σE	
**Stable Plate Interior Sites**												
Delhi DELH	28.48	77.13	Permanent (2003–2005)	35.32	1.25	34.03	1.25	0.67	1.25	−0.92	1.25	Northern most point on stable Indian Plate
Karagpur IITK	26.51	80.23	Permanent (2005–2008)	34.97	1.03	36.78	1.04	−0.49	1.03	−0.26	1.04	Uttar Pradesh
Lucknow LUCK	26.89	80.94	Permanent (2001–2005)	35.44	0.69	37.73	0.70	−0.20	0.69	0.72	0.70	Uttar Pradesh
Lucknow LCKI	26.91	80.96	Permanent (2011–2014)	34.48	0.69	37.37	0.70	−1.16	0.69	0.37	0.70	IGS Station
Lucknow LCK2	26.91	80.96	Permanent (2011–2014)	34.45	0.69	37.70	0.70	−1.19	0.69	0.70	0.70	IGS Station
Varanasi BHUP	25.27	82.99	Permanent (2007–2009)	36.71	1.46	38.65	1.46	0.61	1.46	0.15	1.46	Indo Gangetic Plain
Khavda KHAV	23.92	69.77	Permanent (2010–2011)	31.25	1.47	34.64	1.48	−1.08	1.47	−1.39	1.48	Gujarat
Radhanpur RADP	23.82	71.62	Permanent (2010–2011)	32.49	1.48	38.25	1.49	−0.47	1.48	1.73	1.49	Gujarat
BELA BELP	23.87	70.80	Permanent (2010–2011)	31.80	1.49	34.67	1.49	−0.89	1.49	−1.63	1.49	Gujarat
Mount Abu MABU	24.65	72.78	Permanent (2010–2011)	32.59	1.49	36.31	1.50	−0.75	1.49	0.05	1.50	Gujarat
Dharoi DHAR	24.01	72.85	Permanent (2010–2011)	32.95	1.55	37.34	1.56	−0.42	1.55	0.65	1.56	Gujarat
ISRR	23.16	72.67	Permanent (2010–2011)	33.11	1.53	36.68	1.54	−0.20	1.53	−0.51	1.54	Gujarat
Udaipur UDAI	24.58	73.71	Permanent (2007–2008)	34.83	2.19	37.32	2.21	1.19	2.19	0.78	2.21	Southern part of Rajastan
Bhopal BHOP	23.21	77.45	Permanent (2003–2005)	35.69	1.65	37.22	1.66	0.95	1.65	−1.05	1.66	Madhya Pradesh
Jabalpur JBPR	23.13	79.88	Permanent (2002–2004)	39.02	1.54	40.08	1.56	−0.36	1.54	1.19	1.56	Madhya Pradesh
Dhanbad DHAN	23.82	86.44	Permanent (2004–2005)	36.22	1.59	40.69	1.61	−0.56	1.59	0.52	1.61	Jharkhand
Durgapur DURG	23.53	87.31	Permanent (2007–2009)	37.02	1.33	40.11	1.34	0.09	1.33	−0.42	1.34	West Bengal
Bhubaneswar BHUB	20.26	85.79	Permanent (2001–2012)	37.19	0.63	40.19	0.64	0.52	0.63	−1.54	0.64	Eastern Ghat Mountains, Orissa
Mumbai IITB	19.13	72.92	Permanent (2004–2005)	34.05	1.63	39.14	1.65	0.65	1.63	−0.52	1.65	Maharashtra
Pune PUNE	18.56	73.88	Permanent (2001–2005)	36.67	0.94	39.57	0.94	2.97	0.94	−0.60	0.94	Deccan Plateau, Maharashtra
Hyderabad HYDE	17.42	78.55	IGS (2002–2015)	34.80	0.69	40.65	0.69	−0.25	0.69	−0.99	0.69	IGS Station in Central India located deccan Plateau
Bangalore IISC	13.02	77.57	IGS (1996–2015)	34.97	0.36	42.74	0.36	0.18	0.36	−0.84	0.36	IGS Station in South India Located on Bedrock exposure
Bangalore BAN2	13.03	77.51	IGS (2002–2013)	34.98	0.36	42.73	0.36	0.21	0.36	−0.84	0.36	IGS Station
Kodaikanal KODI	10.23	77.47	Permanent (1998–2015)	34.44	0.53	44.07	0.53	−0.32	0.53	−0.72	0.53	Southernmost point on stable Indian Plate
Trivandrum TVM_	8.42	76.97	Permanent (2002–2005)	35.60	1.20	48.51	1.21	0.97	1.20	3.03	1.21	West Coast of India
Maldives MALD	4.19	73.53	IGS (2000–2006)	35.81	0.86	46.15	0.86	2.21	0.86	−0.74	0.86	IGS station, Southern most of the Indian Plate
Colombo SGOC	6.89	79.87	IGS (2012–2015)	35.23	1.10	44.24	1.11	−0.17	1.10	−2.04	1.11	IGS Station, Narahenpita, Colombo,,Srilanka
**South India episodic/campaign sites**												
Chennimalai CHEN	11.16	77.59	Campaign (1996–2004)	35.73	0.53	44.30	0.53	0.93	0.53	−0.11	0.53	Erode District, Tamilnadu
Palani PLNI	10.43	77.56	Campaign (1996–2004)	35.20	0.53	44.73	0.54	0.41	0.53	0.01	0.54	Tamilnadu
Manaparai MANP	10.66	78.46	Campaign (1996–2004)	35.51	0.53	44.82	0.55	0.48	0.53	0.10	0.55	Salem distrct,Tamilnadu
**Himalaya cGPS Sites from West to East**												
Panamik PAN2	34.71	77.58	Permanent (2004–2005)	22.55	1.53	22.93	1.53	−12.21	1.53	−7.96	1.53	Nubra Valley, NW karakoram Terrane
Leh RSCL	34.13	77.60	Permanent (2002–2012)	21.74	0.68	24.94	0.69	−13.03	0.68	−6.36	0.69	Ladakh
Hanle IAOH	32.78	78.97	Permanent (2001–2015)	19.91	0.56	26.67	0.57	−15.23	0.56	−6.02	0.57	Mt Saraswathi, Tethys Himalaya
Naddi NADI	32.25	76.31	Permanent (2004–2005)	30.38	1.52	24.41	1.52	−4.03	1.52	−7.77	1.52	MBT Zone south of Leh
Kothi KOT1	32.32	77.19	Permanent (2004–2005)	24.44	1.61	25.45	1.61	−10.22	1.61	−6.97	1.61	MCT Zone SW of Leh
GBKL	31.83	77.17	Permanent (2008–2014)	28.22	0.80	26.24	0.81	−6.43	0.80	−6.51	0.81	MCT Zone, On the banks of Beas River
Dehradun DEHR	30.32	78.05	Permanent (2005–2011)	35.91	0.64	33.30	0.65	1.01	0.64	−0.72	0.65	South of MBT in the Lesser Himalaya
Roorkee CBRI	29.87	77.90	Permanent (2014–2015)	32.66	1.73	33.60	1.73	−2.20	1.73	−0.68	1.73	Southwestern part of Uttarakhand
Dehradun WIH2	30.33	78.01	Permanent (2002–2005)	35.53	0.64	32.66	0.65	0.64	0.64	−1.35	0.65	South of MBT in the Lesser Himalaya
Nagoli GBSN	30.20	78.71	Permanent (2008–2014)	31.67	0.81	32.25	0.82	−3.40	0.81	−2.06	0.82	Gharwal Himalaya
Almora GBPK	29.64	79.62	Permanent (2001–2014)	34.68	0.60	33.50	0.61	−0.63	0.60	−1.44	0.61	Lesser Himalaya
Nainital GBNL	29.39	79.45	Permanent (2008–2014)	33.14	0.79	33.41	0.80	−2.12	0.79	−1.64	0.80	Kumaon Himalaya
Munsyari MUNS	30.06	80.24	Permanent (2005)	25.00	2.11	31.40	2.11	−10.46	2.11	−3.47	2.11	Higher Himalaya
DNGD	28.75	80.58	Permanent (2009–2010)	32.73	1.46	34.92	1.47	−2.82	1.46	−0.86	1.47	WesternNepal
Darchula DRCL	29.73	80.50	Permanent (2009–2010)	27.03	1.48	30.00	1.48	−8.49	1.48	−5.15	1.48	WesternNepal
Bhimchula BMCL	28.66	81.71	Permanent (2009–2010)	32.22	1.48	32.95	1.49	−3.59	1.48	−3.23	1.49	WesternNepal
Jumla JMLA	29.28	82.19	Permanent (2009–2010)	25.41	1.46	31.63	1.46	−10.51	1.46	−4.32	1.46	WesternNepal
BYNA	29.47	81.20	Permanent (2010)	23.31	2.10	31.86	2.11	−12.38	2.10	−3.67	2.11	WesternNepal
Dolpa DLPA	28.98	82.82	Permanent (2009–2010)	23.61	1.46	32.02	1.47	−12.45	1.46	−4.30	1.47	Karnali Zone of North Western Nepal
Ghorahi GRHI	27.95	82.49	Permanent (2009–2010)	33.38	1.46	35.07	1.47	−2.61	1.46	−1.76	1.47	South West Nepal
Jomsom JMSM	28.81	83.74	Permanent (2004–2010)	25.73	0.81	35.11	0.81	−10.53	0.81	−1.59	0.81	On the banks of Kali−Gadanki River
Koldana KLDN	27.77	83.60	Permanent (2009–2010)	33.61	1.46	35.49	1.46	−2.62	1.46	−1.77	1.46	Central Nepal
Sarangkot SRNK	28.26	83.94	Permanent (2009–2010)	29.07	1.55	34.20	1.56	−7.23	1.55	−2.88	1.55	Sarangkot Mountain
Simara SIM4	27.17	84.99	Permanent (2004–2005)	37.68	2.04	35.53	2.07	1.17	2.04	−2.47	2.07	South Eastern Nepal
Rumjartar RMJT	27.31	86.55	Permanent (2009–2010)	30.46	1.95	36.34	1.96	−6.33	1.95	−2.04	1.96	Mid Eastern Nepal
Ramite RMTE	26.99	86.60	Permanent (2009–2010)	35.80	1.58	36.31	1.59	−1.00	1.58	−2.26	1.59	South Eastern Nepal
Syangboche SYBC	27.81	86.71	Permanent (2009–2010)	24.48	1.46	36.50	1.47	−12.34	1.46	−1.66	1.47	North Eastern Nepal
Panthang GBSK	27.37	88.57	Permanent (2003–2014)	28.15	0.63	38.28	0.64	−8.98	0.63	−0.67	0.64	Located in Sikkim Himalaya
RBIT	26.85	89.39	Permanent (2003–2005)	35.06	1.42	42.84	1.43	−2.19	1.42	3.39	1.43	Bhutan
TIMP	27.47	89.63	Permanent (2003–2005)	31.65	1.41	41.51	1.42	−5.63	1.41	2.30	1.42	Bhutan
Lhasa LHAS	26.66	91.10	IGS (1996–2007)	16.81	0.44	46.80	0.44	−20.66	0.44	8.27	0.44	IGS Station located in south-eastern Tibet,China
Lhasa LHAZ	29.66	91.10	IGS (2001–2015)	16.81	0.44	46.80	0.44	−20.66	0.44	8.27	0.44	IGS Station located in south-eastern Tibet,China
Bomdilla BOMP	27.27	92.41	Permanent (2004–2013)	20.40	0.67	42.28	0.68	-17.23	0.67	2.14	0.68	Located in Arunachal Himalaya
GBZR	27.59	93.83	Permanent (2010–2014)	15.18	0.99	41.81	1.00	−22.59	0.99	1.40	1.00	Arunachal Himalaya
Anini Anin	28.80	95.90	Permanent (2007–2008)	9.75	1.63	45.58	1.64	−28.19	1.63	5.09	1.64	Arunachal Himalaya
**Northeast cGPS Sites**												
Tezpur TZPR	26.62	92.78	Permanent (2002–2013)	27.90	0.64	40.84	0.66	−9.77	0.64	0.28	0.66	Located in Assam
Guwahati GHTU	26.15	91.66	Permanent (2003–2012)	30.92	0.69	40.71	0.69	−6.63	0.69	0.26	0.69	Located in Assam
Shillong CSOS	25.57	91.86	Permanent (2002–2008)	32.08	0.75	40.58	0.76	−5.49	0.75	−0.21	0.76	Located in Shillong Plateau
Shillong SHIL	25.57	91.88	Permanent (2004–2005)	30.92	0.75	41.04	0.76	−6.65	0.75	0.25	0.76	Located in Shillong Plateau
Lumami LUMA	26.22	94.48	Permanent (2003–2015)	22.13	0.61	38.04	0.62	−15.70	0.61	−3.20	0.62	Indo Burmese Fold and Thrust Belt
Imphal IMPH	24.75	93.92	Permanent (2003–2009)	21.44	0.82	31.54	0.83	−16.35	0.82	−10.19	0.83	Indo Burmese Fold and Thrust Belt
Aizwal AIZW	23.72	92.73	Permanent (2003–2006)	31.06	1.20	34.99	1.21	−6.61	1.20	−6.86	1.21	Indo Burmese Fold and Thrust Belt
**Andaman cGPS site**												
Portblair PBR2	11.64	92.71	IGS (2012–2015)	15.53	1.09	3.58	1.10	−22.15	1.09	−42.55	1.10	Andaman & Nicobar Islands
PBLR	11.61	92.72	Campaign (1996–1999)	28.84	0.50	34.01	1.30	−8.85	0.50	−12.13	1.30	Andaman & Nicobar Islands
PBLC	11.61	92.72	Permanent (2005)	—	—	—	—	−120	1.5	−400	1.5	Post-seismic deformation about 800 days since the earthquake using composite time series of both the cGPS sites
PPBL	11.66	92.74	Permanent (2005–2007)	−	−	−	−					
**IGS Sites**												
Sheshan SHAO	31.10	121.20	IGS (1996–2000)	−17.67	0.63	27.71	0.65	−53.66	0.63	−20.21	0.65	China in Eurasian tectonic plate
Karratha KARR	−20.98	117.10	IGS (1996–2015)	56.96	0.42	41.46	0.41	20.14	0.42	−0.12	0.41	Australian tectonic plate
Wuhan WUHN	30.53	114.36	IGS (1996–2015)	−8.69	0.58	34.10	0.57	−45.93	0.58	−11.74	0.57	China in Eurasian tectonic plate
Bakosurtanal BAKO	−6.49	106.85	IGS (2002–2015)	−9.97	0.75	23.87	0.76	−48.00	0.75	−23.78	0.76	Located in Indonesia
Nanyang NTUS	1.35	103.68	IGS (2001–2015)	−11.71	1.06	22.11	1.07	−49.86	1.06	−26.24	1.07	Located in Singapore
Kunming KUNM	25.03	102.80	IGS (2000–2013)	−18.38	0.67	32.92	0.68	−56.53	0.67	−11.17	0.68	China in Eurasian tectonic plate
Cocos COCO	−12.19	96.83	IGS (1996–2015)	53.86	0.34	42.80	0.34	15.84	0.34	−5.15	0.34	Coco island, western Australian tectonic plate
Selezaschita SELE	43.18	77.02	IGS (2000–2013)	6.32	0.60	27.48	0.62	−28.27	0.60	3.09	0.62	Kazakhstan in Tean Shan tectonic plate
Poligan POL2	42.68	74.69	IGS (1996–2015)	4.43	0.33	27.81	0.33	−29.48	0.33	3.97	0.33	Kyrghyzstan in Eurasian tectonic plate
Diego Garcia DGAR	−7.27	72.37	IGS (1996–2015)	31.50	0.70	48.54	0.71	−1.73	0.70	−1.77	0.71	Diego Garcia Island
Kitab KIT3	39.13	66.89	IGS (1996–2015)	6.51	0.54	27.86	0.55	−24.73	0.54	4.05	0.55	Uzbekistan in Eurasian tectonic plate
Seychelles SEY1	−4.67	55.48	IGS (2001–2015)	10.27	0.95	24.96	0.96	−16.07	0.95	−25.45	0.96	Mahe Island,East African tectonic plate
Bahrain BAHR	26.21	50.61	IGS (1996–2008)	31.35	0.65	30.64	0.62	7.47	0.65	0.32	0.62	Located in Arabian tectonic plate
Bahrain BHR1	26.21	50.61	IGS (2000–2009)	31.61	0.72	28.99	0.69	7.73	0.72	−1.33	0.69	Located in Arabian tectonic plate
Bahrain BHR2	26.21	50.61	IGS (2005–2009)	30.68	0.41	31.30	0.39	6.65	0.41	1.67	0.39	Located in Arabian tectonic plate

**Table 2:** Angular Velocity of Indian tectonic plate motion.

Reference Frame	Latitude (°)	Longitude (°)	Rotation Myr^−^^1^
**India/ITRF2008 Present study**	**51**.**698 ± 0**.**271** RhoLtLg = −0.631	**11**.**853 ± 1**.**790** RhoLtMg = −0.597	**0**.**553276 ± 0**.**005520** RhoLgMg = 0.914
	Wx (deg/Myr) = 0.335613 ± 0.003013; RhoXY = 0.734 Wy(deg/Myr) = 0.070437 ± 0.011401; RhoYZ = 0.817 Wz(deg/Myr) = 0.434185 ± 0.003606;; RhoXZ = 0.620;		
India/ITRF2008^15^	51.4 ± 0.07	8.9 ± 0.8	0.539 ± 0.002
India/ITRF2000^13^	52.97 ± 0.217	−0.297 ± 3.760	0.499 ± 0.008
India/ITRF2000^12^	51.7 ± 0.5	−15.1 ± 1.5	0.469 ± 0.01
India/ITRF2000^11^	51.4 ± 1.6	−10.9 ± 5.6	0.483 ± 0.01
India/ITRF2000^10^	50.9 ± 5.1	−12.1 ± 0.6	0.486 ± 0.01
India/ITRF1997^9^	53.7	−13.9	0.483

Figure 3 shows the residual velocities of the 30 India plate sites with respect to the GPS site velocities that are predicted with our new angular velocity in map view (Fig. 3a) and in velocity space (Fig. 3b). Reduced chi-square, the least-squares misfit normalized by the degrees of freedom, is 1.24. The estimated site velocity uncertainties are thus, on average, nearly equal to the velocity misfits. The weighted root-mean-square (WRMS) misfits to the north and east velocity components are 0.79 and 0.90 mm/yr, with ninety percent of the residual velocities lower than 2 mm/yr (Fig. 3b). The uncertainties in the newly estimated angular velocity are small, partly because it is estimated from more than twice as many continuous station velocities as for any previous study and partly due to the superior geographic spread of those stations in the plate interior relative to previous works.

**Figure 4:**
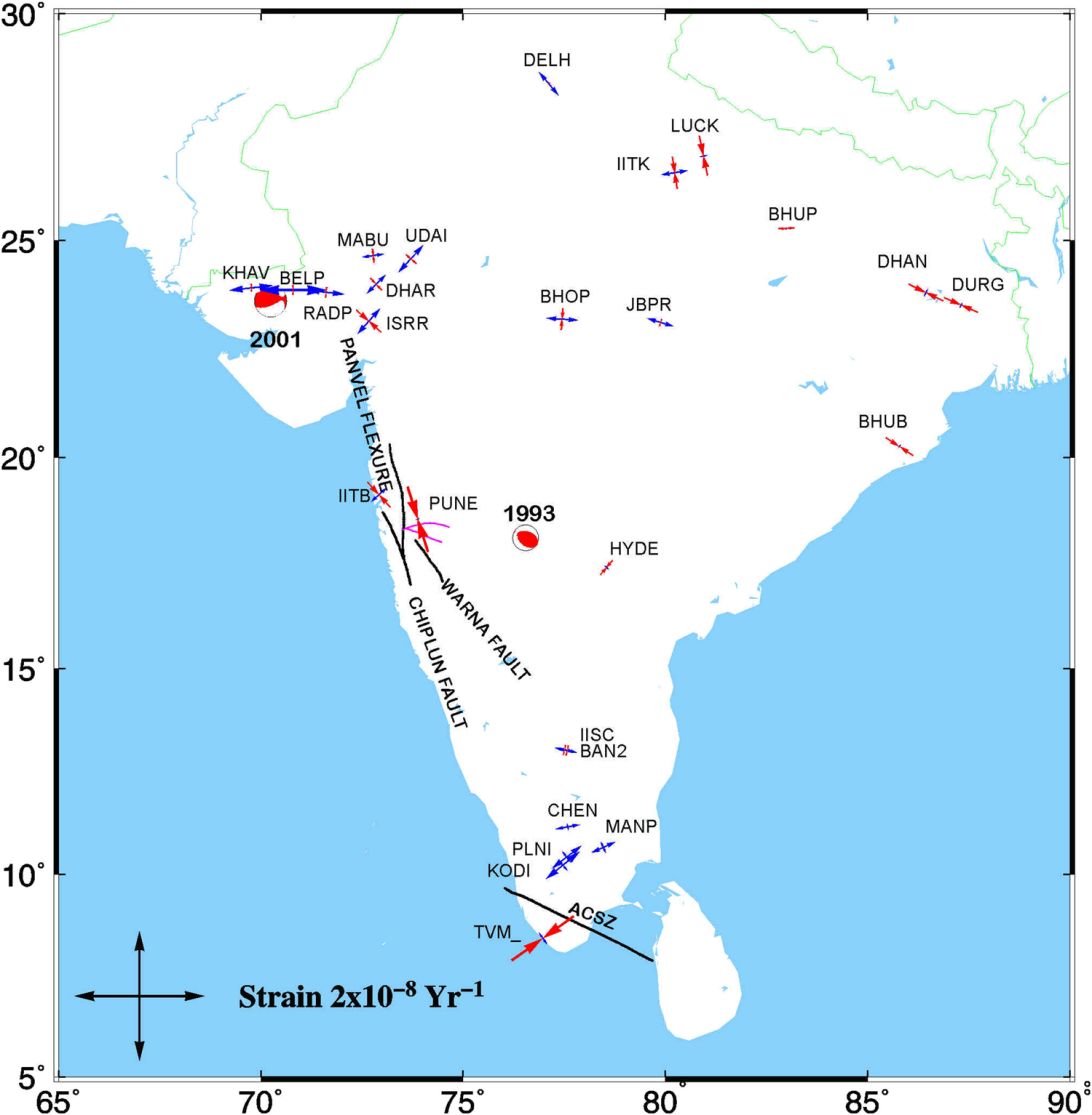
Maximum and Minimum Principal Strains at plate interior GPS sites. Red: Compression, Blue: Extension. ACSZ: Achankovil Shear zone, Blue lines are minor lineaments near PUNE GPS site. Focal mechanisms **(**Source: http://www.globalcmt.org/CMT **)** are given for 2 major earthquakes mentioned in the text, including the 1993 Latur earthquake (Mw 6.3) and the 2001 Bhuj earthquake (Mw 7.6). Figure was created using GMT (generic mapping tool) software^72^.

**Figure 5:**
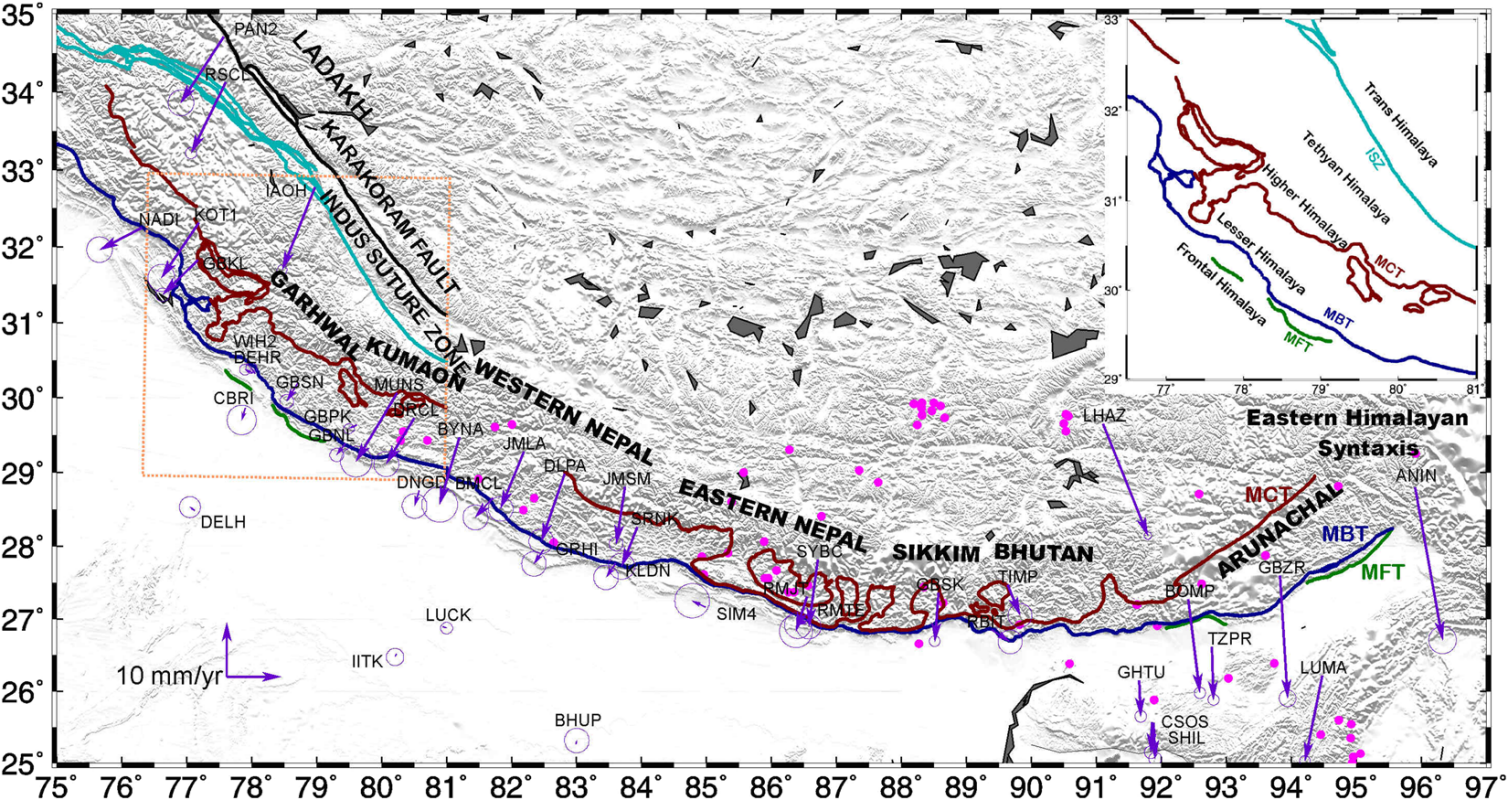
India-fixed velocities tipped with 70% confidence error ellipse of Himalayan cGPS sites along with major fault lines. Green line labelled MFT is the Main Frontal Thrust; blue line labelled MBT is Main Boundary Thrust; dark red line labelled MCT is Main Central Thrust and the light blue labelled ISZ is Indus Suture Zone. Boxed area is zoomed to indicate the Frontal, Lesser, Higher, Tethyan and Trans Himalaya. Earthquakes with magnitude ≥5 from 1970 to 2016 (source: http://ds.iris.edu/) are plotted as solid pink circles. Figure was created using GMT (generic mapping tool) software^72^.

We next describe the motions of all the GPS sites used in the analysis in our newly estimated India plate frame of reference (India-ITRF08) which are given in Table 1. To facilitate the discussion, the deformation is subdivided by region, including continental portions of the plate interior (i.e. peninsular India and the Indo-Gangetic plain) (Figs 3 and 4), the Himalayan Arc (Fig. 5), Northeast India and the Indo-Burmese Arc (Fig. 7) and the Andaman Arc (Fig. 8).

### Plate Interior (4–29°N; 68–88°E)

Peninsular India and the Indo-Gangetic plains north of the peninsula constitute nearly all continental portions of the plate interior, excepting only the island of Sri Lanka (Fig. 3a). The tectonic features of peninsular India are complex with varied geology and numerous, possibly active, but poorly understood faults^35–40^. Since 1900, the level of seismic activity in peninsula (except Kachchh region) can be rated as below moderate to low with very rare occurrence of earthquakes of Magnitude 5.0 and above^29^. Two significant earthquakes have affected this nominally-stable portion of the plate interior in the recent past, the Mw = 6.3 Latur earthquake in 1993^38^ and the Mw = 7.6 Kachchh/Bhuj/Gujarat earthquake in 2001^33, 41^. Although the thrust-faulting focal mechanisms for both earthquakes (Fig. 3a) are consistent with slow ~N-S shortening of the plate interior, the velocities of the mostly continuous GPS sites that span the plate interior show no evidence for an organized deformation pattern at rates faster than ~1 to 2 mm/yr, the approximate resolution of our velocity field. India-fixed composite velocities of peninsular India continuous (except TVM_ and PUNE) and campaign sites are ~0.8 to 1.8 ± 1.5 mm/yr. The Kanpur, Lucknow, Varanasi and Delhi GPS sites on the Indo-Gangetic plain north of peninsular India also indicate composite velocities of ~0.6 to 1.4 mm/yr ± 1.2 mm/yr. Baseline-length changes of the longest North-South baseline KODI–DELH is 1.1 ± 1.3 mm/yr and East-West baseline KHAV-DURG is 1.7 ± 2 mm/yr represent the deformation rates in the continental portion of plate interior.

Strains are computed at all the 28 continental plate interior sites using strain_zero program of Grid-Strain^42, 43^ and plotted in Fig. 4. The maximum principal strain rates for plate interior sites (except TVM_, PUNE and BELP) vary between ~4e-10 to 6e-9 (extension) and the minimum principal strain rates vary from -6e-9 (compression) to 2e-9 (extension) which is consistent with the strain rates reported earlier i.e negligible regional dilatational and shear strain changes in the southernmost 530 km of India^44^; statistically insignificant strain rate of 2.1 ± 6.1 and 2.6 ± 8.4 · 10–9 yr–1 in the north-south and east-west directions respectively^7^; southern peninsula move as a rigid block with the velocity of Indian tectonic plate^45^. The evidence for slow or no deformation in these parts of the plate interior support the hypothesis^15^ that the plate interior is not significantly affected by the Narmada Son failed rift region or any other fault. By corollary, Indian plate motion is well described by a single angular velocity. This study confirms that Indian plate interior region moves as a rigid block with the velocity of Indian tectonic plate with no significant strain accumulation and the occurrence of Intra-plate earthquakes are due to localized regional deformation specific to the active dislocations and faults in the region.

The two isolated cGPS sites TVM_ and PUNE (Fig. 3) indicate ~3 mm/yr India-fixed velocity which cannot be termed as significant in terms of GPS resolution but nevertheless cannot be ignored considering the GPS data span of 3–5 yrs and needs to be taken in to account for further studies in these regions as detailed below. TVM_ cGPS site located in the southern tip of Indian peninsula has India-fixed velocity of ~3.2 ± 1.2 mm/yr NE. This site is located in Achankovil Shear zone which extends 120 km laterally with a width of 20–30 km (Figs 3 and 4). The shear zone strikes NW–SE with distinct rock types to the north and the south^39^. Baseline length between TVM_ and the KODI, nearest cGPS site indicate shortening of 3.7 ± 1.3 mm/yr and the 3 campaign sites (CHEN, MANP, PLNI) north of KODI also indicate shortening of ~2.6–3.6 mm/yr which may be due to the active regional deformation in this shear zone. Strain rate at Trivandrum indicates compression of 1.2e-8 which is consistent with the shortening in this region.This needs further corroboration by dense network of GPS measurements in Achankovil Shear zone.

PUNE GPS site located in the western margin of Deccan Plateau to the north of western ghats of peninsular India indicates India-fixed northward velocity of 3 ± 1 mm/yr in Indian reference frame. Though several faults and lineaments are located in this region (Figs 3 and 4), Panvel flexure is the most conspicuous lineament located in this region which strikes in the north-northwest direction parallel to the west coast and extends approximately between latitudes 16 to 21°N^29^. The origin of Panvel flexure is related to west-coast rifting, subsidence and uplift of Western Ghats. Recent micro-seismic studies indicate existence of active fault systems beneath the Panvel flexure possibly related to west-coast tectonics^40^. GPS motion of ~3 mm/yr in this region and strain rate (compression) of 1.1e-8 may be due to regional deformation related to this active fault system which needs further in-depth study.

The cGPS sites KHAV, BELP, RADP are located in Kachchh rift basin of Gujarat ~50–100 km north of the 2001 Mw = 7.6 Bhuj earthquake epicenter^34^. ISRR GPS site is located in Gandhinagar close to Ahmadabad (~160 km east of epicenter of Bhuj earthquake) and DHAR, MABU are located ~100 and 200 km further north of Ahmadabad. The transient post seismic deformation for Bhuj earthquake is found to be very low and attenuated rapidly within 3–4 years of the earthquake and is much low during 2007–2009 suggesting a weak mantle in this region^46, 47^. India fixed velocities and baseline length changes of these continuous GPS sites located in KRB indicate N-S rate of −0.71 ± 2.1 mm/yr between ISRR -MABU and E-W rate of 2.3 ± 2.11 mm/yr between KHAV-DHAR. These measurements are during 2010–2012 and the residual velocities of 1.8 ± 1.5 mm/yr and strain rate (extension) of ~1e-08 at KHAV and BELP GPS sites located close to the epicenter of Bhuj earthquake do indicate the direction pointing towards the epicenter indicating that there may be visco-elastic effects of the 2001 earthquake which the current resolution of our GPS velocity field is not able to quantify.

### Himalayan Arc

The Himalayan Arc is seismically active between Kashmir in the west and the Eastern Syntaxis in the east (Fig. 5) due to active under-thrusting of India tectonic plate below Eurasian plate^48^. The Himalayan arc can be segmented from west to east into Kashmir, Ladakh, Gharwal, Kumaon, Nepal, Sikkim, Bhutan, Arunachal Himalaya and Eastern Syntaxis. It is characterized by several thrust faults (MFT, MBT, MCT, STD, ISZ) that sole in to the basal detachment i.e MHT which constitutes the surface over which Tibet together with Himalayan wedge moves southward over the Indian plate. From north to south each segment of Himalaya is classified as Trans-Himalaya, Tethyan Himalaya, Higher Himalaya, Lesser Himalaya and frontal Himalaya (Fig. 5) which are bounded by these thrust faults.

**Figure 6:**
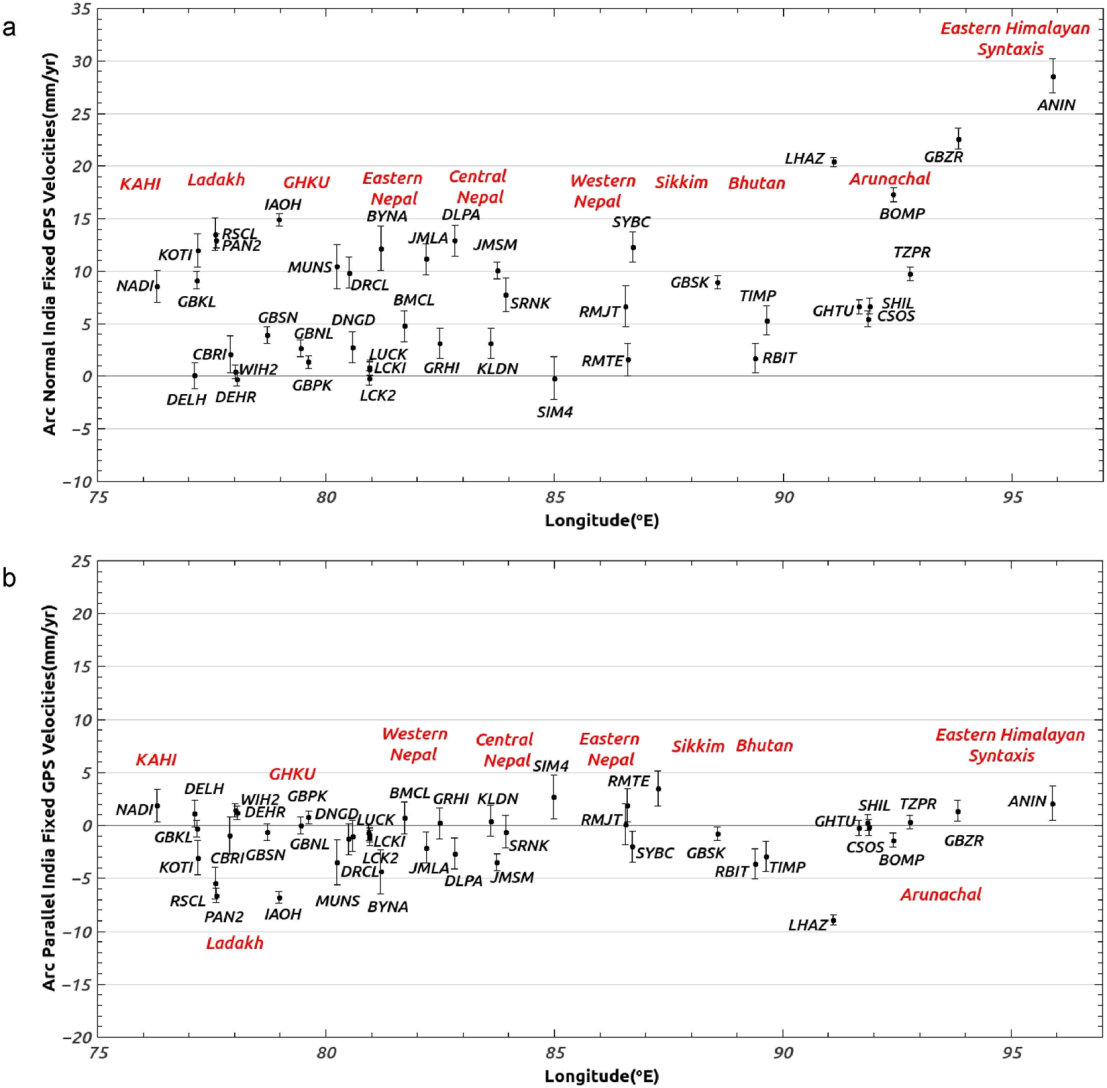
(**a**) Himalaya cGPS site velocity components with error bars orthogonal to the Himalayan Arc in a India-fixed frame of reference. Velocity components were determined by rotating the site velocities on to the direction locally orthogonal to the arc using the arc geometry defined by^74^. Figure was created using qtiplot^73^. (**b**) Himalaya cGPS site velocity components with error bars parallel to the Himalayan Arc in a India-fixed frame of reference. The velocity components were determined by rotating the site velocities onto the direction locally parallel to the arc using the arc geometry defined by^74^. Figure was created using qtiplot^73^.

Deformation parallel and orthogonal to the Himalayan arc is estimated herein with 35 continuous sites, of which 17 are along the Ladakh, Gharwal, Kumaon, Sikkim, Arunachal, and Eastern syntaxis segments of Indian Himalayas, 14 are from the western, central and eastern Nepalese Himalaya, 2 are from Bhutan Himalaya and 2 from South Tibet (Figs 5 and 6). Relative to our newly-estimated India plate frame of reference, the 35 site velocities vary from ~0.2 to 28 mm/yr. Figure 5 shows in map view the velocities of all 35 sites and other sites proximal to the arc and their approximate location from Plate boundary. Figure 6a,b document variations in the velocity components orthogonal and parallel to the Himalayan Arc from west to east. The GPS sites in the foreland immediately south of the arc move southward toward the plate interior at 0.2 to 3 mm/yr, consistent with the elastic effects associated with locking of the Main Frontal Thrust. The arc-normal rates for sites within the Himalayan Arc vary along the arc from west to east and variously move southward at rates of 3–15 mm/yr in the northwestern Himalaya and Nepal Himalaya, ~9 mm/yr in Sikkim; ~6 mm/yr in Bhutan Himalaya; ~16 to 28 mm/yr in Eastern Himalaya (Fig. 6a). The locking depth, width, and slip of Main Himalayan Thrust (MHT) have been estimated previously^11, 24, 49–52^ by modeling the GPS surface deformation rates in different segment of Himalayas. Arc-normal convergence rates reported in this study broadly support the hypothesis of the previous study^24^, that the slip and locking depth of MHT varies for different segments of Himalaya.

Average arc-parallel rate in Himalayan arc is ~3 mm/yr for different segments of Himalaya from Kashmir-Himachal to Arunachal. Ladakh Himalaya cGPS sites to the west and Lhasa IGS site in south Tibet to the east indicate average arc-parallel extension rate of ~6 to 9 mm/yr which is broadly consistent with the E-W GPS extension rates reported in Tibet^24, 53^. Spatially non-uniform E–W extension of ~9.7 mm/year that occurs between 78°E and 92°E in ~N110°E direction across the sub-structures in southern Tibet was reported earlier^49^. Their model also suggests that a additional ~3 mm/year deformation over the same distance and direction results from the curvature of locked MHT. Three-dimensional block model of the greater Tibetan Plateau region (20–45°N; 76–108°E) was given^54^ by taking into account the inter-seismic GPS deformation, mapped fault geometry, earthquake cycle and moment release. The arc-parallel extension rates in 2500 km Himalayan arc (Fig. 6b) reported here from 35 cGPS sites support this hypothesis^49, 54^.

### Northeast India and the Indo-Burmese Arc

**Figure 7:**
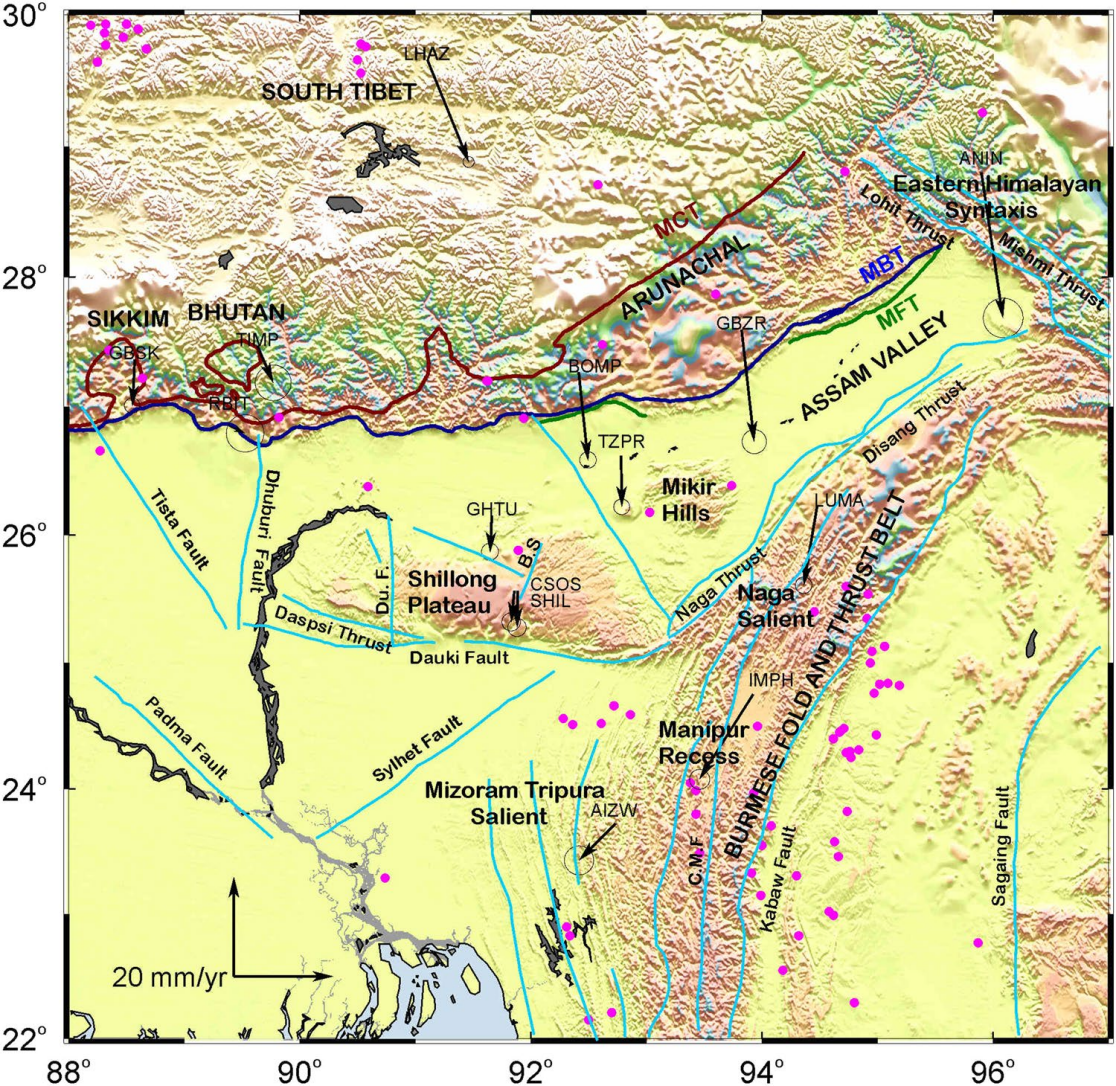
Velocities tipped with 95% confidence error ellipse of GPS sites in northeastern India and the Indo Burmese Arc in an India-fixed frame of reference. All the major fault lines and tectonic domains are marked in the Figure. Lhasa IGS site located in south Tibet is also shown in the Figure. Solid pink circles locate earthquakes with magnitude ≥5 from 1970 to 2016 (source: http://ds.iris.edu/). Figure was created using GMT (generic mapping tool) software^72^.

This seismically active and tectonically complex region is bounded by two convergence plate boundaries i.e Himalayan Arc and Indo-Burmese Arc (Fig. 7). The distinct tectonic domains of northeast India are elevated Shillong Plateau, Brahmaputra (Assam) valley, Indo-Burmese Arc, Eastern Himalaya (Sikkim, Bhutan, Arunachal) and Assam/Eastern Syntaxis. This region has several faults (Fig. 7), with some of the faults being reported to be active. India –fixed velocities of Shillong plateau and Assam valley cGPS sites during the study period indicate southward motion of ~7 mm/yr with respect to the stable Indian shield which is consistent with the rates reported earlier^12, 55^. Campaign GPS measurements^55, 56^ indicate clockwise rotation of Shilling plateau and Assam valley which they attribute to their locations between the Indo-Eurasian and India-Sunda convergence zones. The cGPS site, TZPR in Assam Valley (Fig. 7) moves ~3 mm/yr southward relative to cGPS sites CSOS/SHIL and GHTU in the Shillong Plateau and Assam Valley. The motion between these sites is attributable to the slip along Kopili fault which fragments the Assam Valley and is consistent with previously reported rates^15, 55^.

The Indo-Burmese Arc (IBA) is the western extension of eastern boundary of Indian plate, which is defined by dextral Sagaing fault and Sunda-Arakan trench. This region is segmented by several transverse/oblique faults and has salient-recess topography consisting of foreland Tripura salient in the south and Naga salient in the north which are connected by Manipur recess^12^. India-fixed velocities (Fig. 7) of the three cGPS sites (LUMA, IMPH, AIZW) located in the salient-recess of Indo-Burmese Arc indicate SSW motion of ~16 mm/yr at the northern most site LUMA (Naga Salient); SW motion ~19 mm/yr at IMPH (Manipur Recess) site and ~9.5 mm/yr SW motion at the southernmost site AIWL (Tripura Salient). Indo-Burmese Arc is accommodating about half of the 36 mm/yr relative motion between the India and Sunda plates^10, 57–59^. Baseline lengths between these sites indicate extension of ~3 mm/yr between LUMA and IMPH and shortening of ~9 mm/yr between IMPH and AIZW indicating segmented deformation related to the salient-recess topography and faults in this region^12, 55^. An approximately E-W baseline (GHTU-LUMA) between Shillong Plateau and IBA indicates shortening of ~3.6 mm/yr in this region which is due to the subduction of IBA. The cGPS station ANIN northeast of the Lohit thrust fault in the Eastern Himalayan Syntaxis (Figs 6a and 7) indicates ~20 mm/yr oblique convergence between Indian and Eurasian plate and ~8 mm/yr convergence to the south of frontal Himalaya as indicated by the southward motion of CSOS, SHIL, GHTU, TZPR and LUMA in Shillong Plateau, Assam Valley and IBA indicating that faults in this region accommodate 30% of the ~28 mm/yr convergence rate in this region.

### Andaman Arc

**Figure 8:**
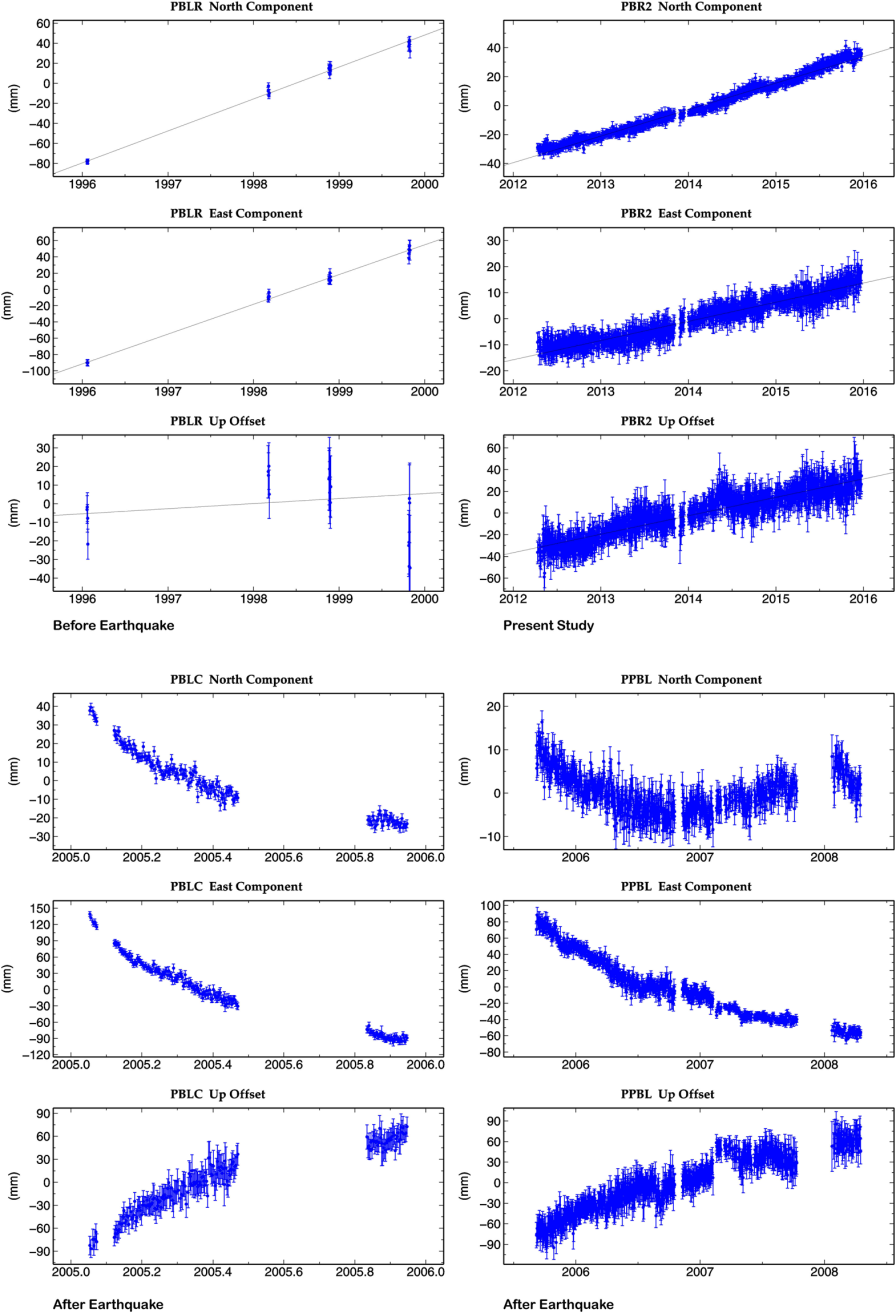
N, E, U positional time series of Port Blair Andaman campaign (1996–1999) site before the 26 December, 2004 Sumatra earthquake of Mw 9.3 and Port Blair cGPS sites after the earthquake PBLC (2005), PPBL (2006–2008) and PBR2 IGS site (2012–2015) of present study. Port Blair site moved southwest by ~3.5 ± 0.01 meters and experienced subsidence of 0.9 meters during the 2004 mega event. Figure was created using GMT (generic mapping tool) software^72^.

GPS data of ~four years (2012–2015) from the Port Blair IGS site (PBR2) in the Andaman Arc (Fig. 2, Table 1) to south of the IBA give an India-fixed velocity of 47.9 ± 1.1 mm/yr toward S62°W (i.e ITRF 2008 velocity of 15.5 ± 1.1 mm/yr N and 3.6 ± 1.1 mm/yr E). Figure 8 gives the position time series of PBR2 in north, east and up direction for ~4 years. For comparison time series of the north, east, up component from our earlier analysis were plotted for four epochs of episodic GPS measurements made at Port Blair before earthquake (1996–1999) and at two cGPS sites in Port Blair soon after the earthquake (2005–2008) in Fig. 8. Inter-seismic motion measured from 1996 to 1999 at a nearby episodic site in Port Blair (Fig. 8) averaged 29.7 ± 1 mm/yr N and 33.3 ± 1.5 mm/yr E in ITRF2005^7, 45, 60^, i.e approximately 15 mm/yr toward S54°W in an India-fixed reference frame. The station velocity during the years before the 2004 Sumatra earthquake was thus only ~one-third as fast as the 2012–2015 post-seismic velocity. During the Mw 9.3 Sumatra 2004 earthquake this region experienced co-seismic motion of ~3.5 ± 0.01 m SW and subsidence of 0.9 m^61^. This region recorded a cumulative post-seismic deformation of ~12 cm S and ~40 cm W and ~24 cm uplift till February 2007 about 800 days since the main event^60^ and the temporal variation of the displacement suggests logarithmic decay (Fig. 8). Time series of PBR2 site (Fig. 8) during 2012 to 2015 do not indicate logarithmic decay of displacement observed during 2005–2007 period^60^. PBR2 site records ~16 mm/yr motion in the up direction during ~4 year period.

## Conclusions

We estimate a new angular velocity for the India plate in ITRF2008 (Table 2) from the velocities of 27 continuous and 3 long-term episodic sites well distributed in the stable plate interior, including the offshore Srilanka and Maldives IGS sites. Our new India-ITRF2008 pole is located ~3 degrees east of the previous estimate^15^, with a slightly (~3%) higher angular velocity considering that both the poles were estimated using ITRF2008 velocities. This difference may be attributed to using 30 GPS sites, more than any previous study and also for the first time GPS sites from the west and east of the plate interior were used in the angular velocity estimate as well as long span of data. Velocity residuals are less than 2 mm/yr for most of the sites located in Indian Plate interior. Strain rates at the continental plate interior sites give maximum extension and compression rates of ~6e-09 indicating that there is no segmentation of the Indian plate along failed rifts or faults and the plate motion is best described with a single angular velocity. Intra-plate earthquakes are due to localized deformation along major faults in the plate interiors which does not contribute to the Indian plate motion.

In the Indian reference frame, TVM_ site in Achankovil Shear zone moves ~3 mm/yr N72°E and PUNE site in Panvel flexure zone moves ~3 mm/yr northward both the sites indicating strain rate (compression) of 1e-08 which may be related to localized active deformation along major faults in these region. This needs further investigation with dense network of GPS measurements to delineate the regional deformation in these regions. KHAV and BELP cGPS near the epicenter of Bhuj earthquake record maximum extension of ~1e-08 compatible with a post-seismic viscoelastic response to the 2001 earthquake which could not be quantified by the current resolution of GPS velocity field.

For the first time arc-normal and arc-parallel rates were estimated using 35 cGPS sites covering the entire 2500 Km Himalayan arc from west to east. Himalayan foreland sites move 0.2–3 mm/yr southward and the rest of the sites move southward at a rate of 3 to 28 mm/yr along the main thrust zones of the different segments of Himalayas i.e NW Himalaya (~15 mm/yr), Sikkim Himalaya (~9 mm/yr), Bhutan Himalaya (~6 mm/yr), Arunachal Himalaya (~20 mm/yr) to maximum rate (~28 mm/yr) in Assam syntaxis. In addition, this total convergence in each segment is accommodated differently in Lesser, Higher, Tethyan and Trans Himalaya. Average arc-parallel motion of ~3 mm/yr recorded in the Himalayan arc is a result of curvature of locked MHT. Arc-parallel extension rate of ~9 mm/yr recorded by Ladakh cGPS and Lhasa site located in South Tibet is due to the extension of Tibet Plateau.

Shillong Plateau and Assam Valley in Northeast India behaves as a distinct block moving southward at a rate of ~7 mm/yr with reference to the plate interior and consistent with the clock wise rotation of this block reported earlier^55^. Shortening and extension rates recorded by cGPS sites in Shillong Plateau, Assam Valley and IBA are consistent with the fragmentation of Assam Valley by Kopili fault and segmented deformation related to the salient-recess topography and faults in Indo Burmese Arc^12, 55^. To the south of IBA, Port Blair site in the Andaman subduction zone to the east of Indian plate moves ~48 mm/yr SW with ~16 mm/yr uplift during ~4 year period (2012–2015), thus defining the upper bound of the ongoing active post-seismic relaxation in this region. Indian plate boundary in north and the east is seismically active^62–67^ with cluster of seismic events (M < 5) recorded along the MCT, IBA and Andaman Arc which indicate that the stress accumulated due to the present-day active deformation in this region is being intermittently released by these events. Inverse modeling of these GPS derived deformation rates at the plate boundaries when combined with active seismicity and seismic gaps of the region give valuable information on earthquake hazard estimation.

### Methodology

#### GPS Data and Analysis

Continuous data from 44 Indian GPS sites established as part of Indian GPS network, episodic GPS data from 3 sites in southern India (spanning 17 years), 16 cGPS sites in Nepal and Bhutan Himalaya, and the Indian IGS station data were analysed for the period 1996 to 2015 using GAMIT/GLOBK and the IGS stations shown in Fig. 1. Detailed information about the sites and data used is given in Table 1. Quality control on all RINEX observation files was done with TEQC software^68^. Observation sessions shorter than 12 hours or with numerous cycle slips or poor multipath statistics were eliminated from the analysis. Phase data of all GPS stations were analyzed following the methodology^69^ using GAMIT/GLOBK software to obtain loosely constrained daily station positions^69, 70^. A data sampling interval of 30 s and elevation cut off angle of 15° was used for all GPS sites. Loosely constrained, daily site positions from GAMIT were refined by minimizing the errors contributed by satellite and receiver clock errors, phase ambiguities, atmosphere, receiver phase center variations and multipath. Zenith tropospheric delays were estimated every two hours using a piecewise linear (PWL) model with 0.5-m priori constraints in zenith delay. Ambiguity-free and ambiguity-fixed solutions were performed with the ionosphere-free linear combination to account for carrier phase ambiguities and signal delay due to the ionosphere. Daily loose GAMIT solutions with their covariance matrix were combined using GLORG for reference frame definition by stabilizing the IGS sites to their ITRF08 coordinates and velocities. Errors in modeling the orbits and atmosphere in the long time series and time-correlated sources of errors in position estimates including monument instability were accounted for by including random-walk noise of 2 mm/yr in the station coordinate time series during the reference frame stabilization. Table 1 gives the ITRF2008 velocities and their uncertainties from our analysis. The same velocities and their associated error ellipses are shown in Fig. 2.

#### Strain Estimation at Plate interior sites

Crustal strain rates are estimated from India-fixed GPS velocities of plate interior sites using modified least-squares (MLS) approach^71^ and coded in MATLAB as grid-strain program^43^. At any GPS site P surrounded by N GPS sites whose positions and displacements are x_n_ and u_n_, n = 1, 2, … N, the functional model for Least Square is u = AI + e where matrix A contains the information related to positions of GPS points, I is the strain vector and e is the residual vector. In the MLS approach, computations are performed emphasizing the effect of nearest sites based on adjustment of least-squares covariance matrix by using a scale factor. This gives the choice to use different scales of analysis and evaluate the scale-dependent behaviour of the observed system and select a scale factor that best fits the observed system. We have used the strain_zero program which is a simplified version of grid-strain to calculate strain at GPS points with a scale factor of 150 which best fits the deformation of plate interior sites. This approach gives an option to reduce or exclude a particular GPS point for strain computation which facilitate in estimating the strains of unevenly distributed cluster of GPS points. Maximum and minimum principal strains and the directions thus estimated for the continental plate interior sites are plotted in Fig. 4.
